# Effective Pneumothorax Detection for Chest X-Ray Images Using Local Binary Pattern and Support Vector Machine

**DOI:** 10.1155/2018/2908517

**Published:** 2018-04-03

**Authors:** Yuan-Hao Chan, Yong-Zhi Zeng, Hsien-Chu Wu, Ming-Chi Wu, Hung-Min Sun

**Affiliations:** ^1^Institute of Information Systems and Applications, National Tsing Hua University, Hsinchu, Taiwan; ^2^Department of Computer Science and Information Engineering, National Taichung University of Science and Technology, Taichung, Taiwan; ^3^Department of Computer Science and Information Engineering, National Chin-Yi University of Technology, Taichung, Taiwan; ^4^School of Medicine, Chung Shan Medical University, Taichung, Taiwan; ^5^Research Center for Information Technology Innovation, Academia Sinica, Taipei, Taiwan

## Abstract

Automatic image segmentation and feature analysis can assist doctors in the treatment and diagnosis of diseases more accurately. Automatic medical image segmentation is difficult due to the varying image quality among equipment. In this paper, the automatic method employed image multiscale intensity texture analysis and segmentation to solve this problem. In this paper, firstly, SVM is applied to identify common pneumothorax. Features are extracted from lung images with the LBP (local binary pattern). Then, classification of pneumothorax is determined by SVM. Secondly, the proposed automatic pneumothorax detection method is based on multiscale intensity texture segmentation by removing the background and noises in chest images for segmenting abnormal lung regions. The segmentation of abnormal regions is used for texture transformed from computing multiple overlapping blocks. The rib boundaries are identified with Sobel edge detection. Finally, in obtaining a complete disease region, the rib boundary is filled up and located between the abnormal regions.

## 1. Introduction

Medical imaging refers to the technical and processing of noninvasive acquisition of internal tissue images of a part of the human or human body for medical or medical research. It is a kind of inverse reasoning calculation. As a science, medical images belong to biological images and include imaging diagnostics, radiology, endoscopy, medical thermography, medical photography, and microscopes. In addition, techniques such as brainwave mapping and magnetoencephalography, although focusing on measurement and recording, do not show significant images, but because of the locating characteristics (i.e., containing positional information) of the resulting data, it can be considered as another form of medical image.

In clinical application, also known as medical imaging, or imaging medicine, some hospitals will have to have a medical imaging center, medical imaging department, or medical imaging department; set up related equipment; and prepare for dedicated nurses, radiologists, and physicians, responsible for the operation of instrumentation, interpretation, and diagnosis of images, which is different from radiation therapy for radiology.

In medical science, medical engineering, medical physics, and biomedical information science, medical imaging usually refers to the science of researching and developing images and capturing and storing technologies and equipment. Research on how to interpret and diagnose medical images is a supplementary science belonging to the radiology department or other medical fields (e.g., neurology, cardiovascular diseases).

Digital image processing has been widely applied in the medical domain. However, most of the methods still require manual processing. Automatic image segmentation and features analysis can assist doctors in the treatment and diagnosis of diseases with higher accuracy, accelerate diagnosis process, and improve efficiency. Automatic medical image segmentation is difficult due to the varied image quality caused by equipment and dosage. In this paper, the automatic method employed image multiscale intensity texture analysis and segmentation to solve this problem. The proposed methods automatically recognize and classify abnormal region without manual segmentation. Generally, automatic identification is based on the difference of textures and organ shapes, or any pathological changes of the lung area. Therefore, the important features could be retained to identify abnormal areas.

In this paper, the chest X-ray images are utilized for identifying lung-related health issues. The first proposed identifying common pneumothorax classification method is based on SVM. Features are extracted from the lung image by the local binary pattern. Then, classification of pneumothorax is determined by support vector machines. The second proposed automatic pneumothorax detection is based on multiscale intensity texture segmentation. The background and noises in the chest images are removed for segmenting the abnormal lung region. Since the area of rib boundaries can be affected easily, the rib boundaries are identified by Sobel edge detection. Finally, in order to cover the complete disease region, abnormal regions are located.

Medical imaging serves as an important source of information for anatomy and organ function along with the diagnosis of diseases. The integration of image processing techniques to medical imaging with machine learning methods has led to the development of computer-aided diagnostic and decision-making tools. The X-rays have the advantages of rapid reproducibility and low cost.

Among them, pneumothorax is a common and easy relapse disease in the diagnosis of chest X-rays. The pneumothorax is an abnormal accumulating of air in the pleural space and leads lung discrete from the chest wall [1, 2]. In general, symptoms range from asymptomatic to life-threatening respiratory distress. If the pneumothorax is significant, it can cause a shift of the mediastinum and compromise hemodynamic stability. There are several types of pneumothorax: primary spontaneous pneumothorax, secondary spontaneous pneumothorax, and iatrogenic/traumatic pneumothorax. However, conditions such as smoking, a common cold, and a family history of pneumothorax may cause spontaneous pneumothorax. On the other hand, the blunt trauma, penetration trauma, and surgery would result in traumatic pneumothorax. According to the United States research, pneumothorax is prevalently occurring in humans, and its relapse ratio is 35% in men [3]. Furthermore, critical pneumothorax may lead to hypoxia, shock, and even death [4].

**Figure 1:**
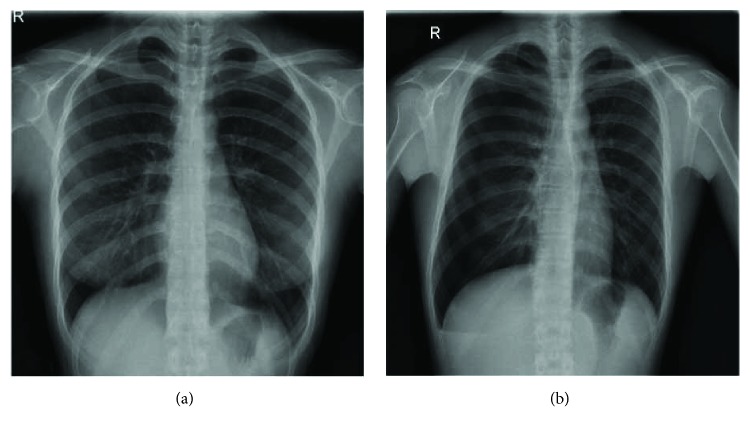
Chest radiographs of (a) normal and (b) pneumothorax.

The chest radiograph has been used as a general adjunct for pneumothorax screening and diagnosis. According to ionizing radiation form of X-rays, the chest images have been generated with the absorption of different spectrums based on the tissue density [5]. Chest radiograph scans the whole chest and produces abundant information and radiologists have to review the data [6]. However, the pneumothorax may be missed or misclassified as other diseases easily in using chest radiographs, because pneumothorax has the characteristics of curved contour and smooth regions within dark region against the chest wall and ribs, and clavicles may overlap [7]. Previous work [7] that proposed the method is based on the image processing gradient-histogram analysis, and its use of many methods is quite complicated, and even many steps that require manual adjustment of parameters in the patent did not put forward the prediction of pneumothorax picture accuracy. This study mainly uses SVM, LBP, Sobel edge detection, and other methods to automate small sample prediction and achieve 85.8% accuracy on patch size 5 × 5 blocks. If clinicians use the method proposed in this study, there is considerable confidence in automation and efficiency. Examples of abnormality for normal chest and pneumothorax are shown in Figure 1.

In the analysis of radiographs, the doctor diagnosed pneumothorax mainly through the main observation of the affected area will have a visceral pleural line, the outer periphery of the pleura line because of the gas, so in the image cannot be seen by the pulmonary blood vessels constitute lung markings and mediastinal displacement, defined in the British Thoracic Society guideline as chest wall larger than 2 cm for large pneumothorax and less than 2 cm for small pneumothorax.

The advantage of X-ray is the fast and inexpensive imaging, and experienced physicians can quickly identify the pneumothorax from the images. In this study, the methods used by these physicians were considered expert knowledge to serve as the machine learning direction for image processing.

The computer-aided diagnosis (CAD) has the potential for improving the diagnostic accuracy of radiologists to distinguish between pneumothorax and other conditions [8]. Several automated analysis of chest radiograph image algorithms had been developed [9]. Geva et al. [10] proposed a visual bag-of-words representation for classification of chest radiograph with different pathologies. Based on the local and global texture signature scheme, the chest radiograph was used to generate localized texture analysis for the detection of local abnormalities. The method applied training and detection of the abnormality by using Gentle AdaBoost to classify normal and abnormal images suggested by Geva et al. [10].

Previous researchers were mostly developed for the diagnosis of the chest radiograph, not for image segmentation and detection for abnormal location. These methods, which concentrate on diagnosis, are viable only on significantly different diseases. Moreover, the location of abnormal region detection is necessary for evaluating treatment guidelines of symptoms and acute disease indicators. For the purpose of detecting the location of abnormal regions, this paper proposed an image segmentation based on texture and intensity distribution for the chest radiograph. The method uses the texture analysis based on intensity and gradient for region segmentation to find out the pneumothorax regions.

At present, there are skin cancers and tuberculosis on the automatic recognition of machine learning or depth learning algorithms. This research focuses on pneumothorax machine learning classification. At present, there is no literature that has focused on pneumothorax medical image classification. The reasons for choosing Sobel edge detection is that it calculates very fast and efficiently on the edge of the medical images. This study uses SVM for its high classification efficiency and deals with high-dimensional spatial data effectively.

## 2. Identifying Pneumothorax Using Machine Learning Algorithms

The SVM-based identifying common pneumothorax method using local binary pattern is explained step by step as follows.

### 2.1. The Proposed Scheme

**Figure 2:**
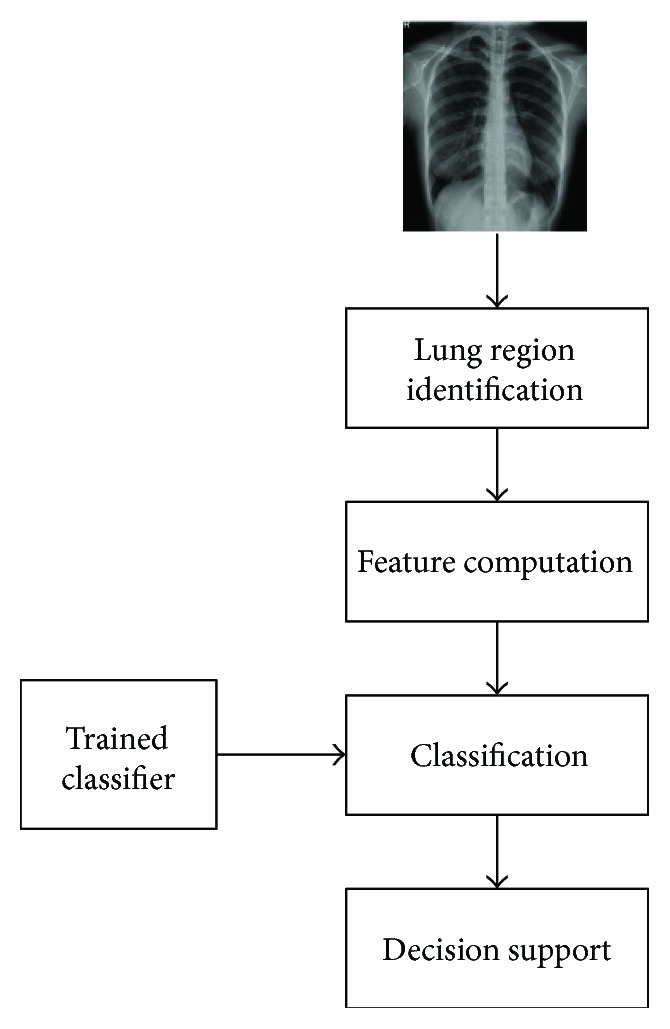
The flowchart of the proposed SVM-based lung classification.

This paper presents identifying common pneumothorax system consisting of lung object identification, feature computation, and training and classification. Lung region identification processes the original image to determine the mean intensity of global lung regions. Feature computation processes the multiblock uniform local binary pattern (ULBP) to calculate each pixel of lung regions and count on the histogram. Finally, support vector machines train and classify normal lungs from abnormal lungs using ULBP features. The flowchart of the support vector machine- (SVM-) based common pneumothorax identification is shown in Figure 2.

### 2.2. Identification of Lung Regions

**Figure 3:**
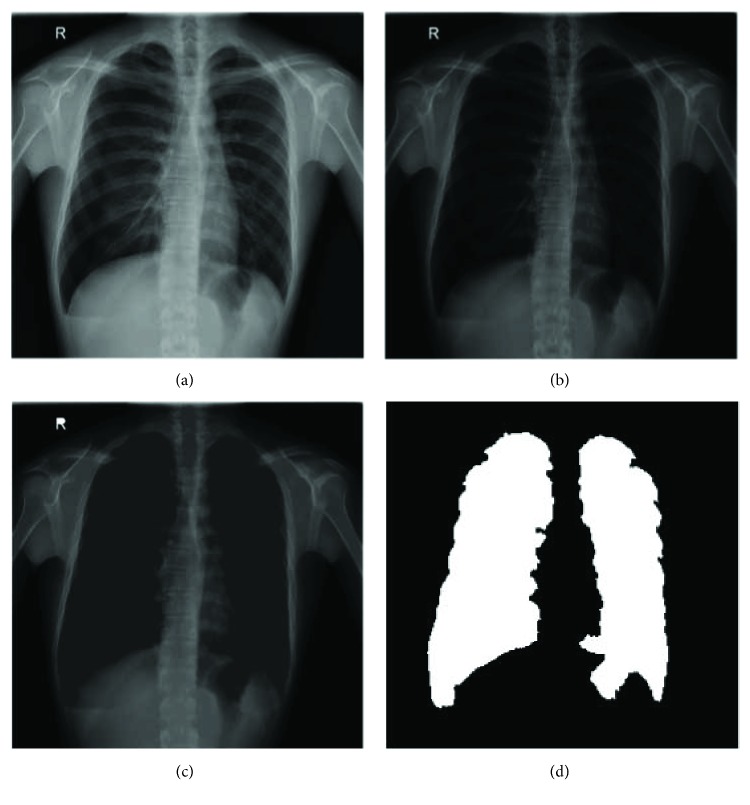
The results of an example (a) original image, (b) the enhanced image, (c) enhanced by hole-filling image, and (d) image lung region identification.

The lung region identification is used to segment the objective global lung regions from original X-ray chest image. After image loading, the mean intensity values of the original image have to be enhanced to the range of intensity from 0 to 1 based on gamma correction to provide useful image intensity details through adjustment of the gamma value [11]. In this process, we assign normalized gamma value to be 2, because the normalized gamma function can use the pixel intensity to provide multiequalization darkness preservation. Consequently, the higher gamma parameter generates a more significant adjustment to display the lung region. The enhancement formula is described as follows.

(1) $$\begin{array}{c} enh\left(h,w\right)={\left(\frac{I\left(h,w\right)}{\mathrm{Max}\_graylevel\_num}\right)}^{\gamma }, \end{array}$$

where *I*(*h*, *w*) is the pixel value of the original image, Max_graylevel_num is the number of the original intensity range, and *γ* is the gamma value.  Figure 3(a) shows an example of original image, and Figure 3(b) shows enhancement result. Because several lighter pixels surrounded by dark pixels lead to generate holes of intensity pixels in the enhanced image, the connect-component method is applied to fill the holes in the lighter pixels surrounded by dark pixels [12].

Finally, the enhanced image defines the integrated lung regions via the Otsu algorithm [13] and transforms into a binary image, and then the lung regions are identified based on 8-connected neighborhood method to find two maximum objects that are able to fill the empty spaces within the regions; Figure 3 shows an example.

### 2.3. Local Binary Pattern Feature Extraction

**Figure 4:**
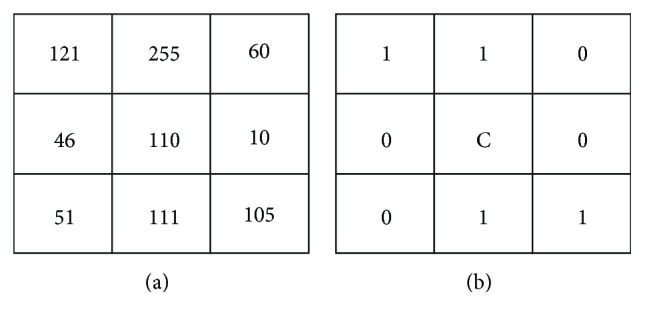
An example of LBP. This result values encoded as 11001100 by starting from the upper left and reading clockwise.

By following the lung region identification, local binary pattern (LBP) is the primary technique in the section. LBP is a local texture operation technique that is considered to be a simple and efficient texture operator. LBP operator uses the center as thresholding for each pixel and compares with neighbor pixel, then the results transformed into the value of binary. Each result of binary value is counted on a histogram. For example, original values are shown in Figure 4(a) in the 3 × 3-sized block.  Figure 4(b) uses the center as the threshold in the 3 × 3-sized block. Finally, each 8-bit LBP is counted on 256-bin histogram.

In addition, LBP operator could adopt the uniform patterns to reduce the length of the feature vector and implement rotation invariant descriptor in a simple way. In this section, the features are calculated through a uniform local binary pattern (ULBP). Uniform pattern is contained at most two bitwise transitions from 0 to 1. For example, the patterns 00000000 (0 transitions), 01111100 (2 transitions), and 11001111 (2 transitions) are uniform. And the patterns 11001101 (4 transitions) and 01010110 (6 transitions) are not uniform. Computation of the ULBP histogram has a separate bin (e.g., the pattern 01000000 and the pattern 00100000 have the same bin).

There are 8 bins for each combination with no more than two conversions (e.g., 10000000 > 01000000 … > 00000001, 11,000,000> 01100000 > … > 0000001111100000> 01110000 > … > 00000111), and each will have 8 patterns for every uniform pattern. And all nonuniform patterns are assigned to a single bin. ULBP image is constructed by *d × d* size. ULBP operator is used for overlapping and scanning lung regions, then generates pattern histogram from the lung image for overlapping and scanning lung regions in lung identification. The neighboring correlation of pixel pattern histogram after encoding transformation in a moving window is defined as

(2) $$\begin{array}{c} X_{n}=\overset{h}{\underset{i=1}{\sum }}\overset{w}{\underset{j=1}{\sum }}H\left\{ULBP\left(i,j\right)==k\left(n\right)\right\},\;n=1,2\ldots ,58, \end{array}$$

where *X_n_* is the number of the *n*th bin in the histogram. *h* is the height of the lung image and *w* is the width of the lung image. *k* is a set, which includes 58 uniform binary patterns corresponding to the integers 0, 1, 2, 3, 4, 6, 7, 8, 12, 14, 15, 16, 24, 28, 30, 31, 32, 48, 56, 60, 62, 63, 64, 96, 112, 120, 124, 126, 127, 128, 129, 131, 135, 143, 159, 191, 192, 193, 195, 199, 207, 223, 224, 225, 227, 231, 239, 240, 241, 243, 247, 248, 249, 251, 252, 253, 254, and 255. *i* and *j* are the pixel coordinates of the lung image. *H*{*S*} is equal to 1 if *S* is true, and *H*{*S*} is equal to 0 if *S* is false. *H* is the part of the histogram and *n* is the value of bin that judging (*i, j*) as the center of the ULBP value is equal to which bin in the histogram corresponding position accumulation

(3) $$\begin{array}{c} ULBP\left(i,j\right)=\min\left(\begin{array}{c} ROT\\ 0\leq b<8 \end{array}{\left[LBP\left(i,j\right)\right]}_{b}\right). \end{array}$$

**Figure 5:**
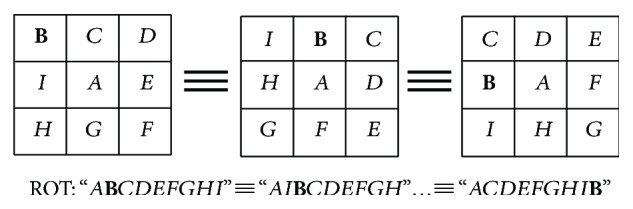
The ROT function, as the same type in the case of clockwise rotation.

ROT is a function fixed with the central pixel, while the neighbors are circularly rotated; *b* is the number of *b*th rotations. An example is shown in Figure 5.

(4) $$\begin{array}{c} LBP\left(i,j\right)=\overset{d}{\underset{k=0}{\sum }}2^{k}Win\left(x_{k}-x_{c}\right),\\ Win\left(x_{k}-x_{c}\right)=\left\{\begin{array}{ll} 1, & \text{if }x_{k}\geq x_{c},\\ 0, & otherwise, \end{array}\right\} \end{array}$$

where LBP is a function value where *x_c_* is a center of the *d* × *d*-sized block, and *x_k_* is the neighboring pixel. Win is a binary thresholding function. Calculate the LBP straight: *x_c_* as the center point with the adjacent point *x_k_* comparison; if greater than or equal to the adjacent point, set to 1 and vice versa 0.

### 2.4. Machine Learning Algorithm

After feature computation, the classification of common pneumothorax is determined by support vector machines. Generally, classification includes input data and divides into training and testing sets. The training set has one label value (class labels) in each lung image in which normal lung image is set to 0, otherwise is set to 1. And each lung image has multiple attributes (features). The attributes are equal to each bin value on ULBP histogram from the feature computation. The features obtained 118 dimensions by using ULBP operator with the 9 *×* 9-sized block and 11 *×* 11-sized block. These constitute the training vectors through the use of the RBF kernel function. Finally, SVM constructs a model classification for normal lung or abnormal lung.

## 3. Rapid Pathology Detection for Chest X-Ray Images Using Texture Segmentation Method

### 3.1. The Proposed Scheme

**Figure 6:**
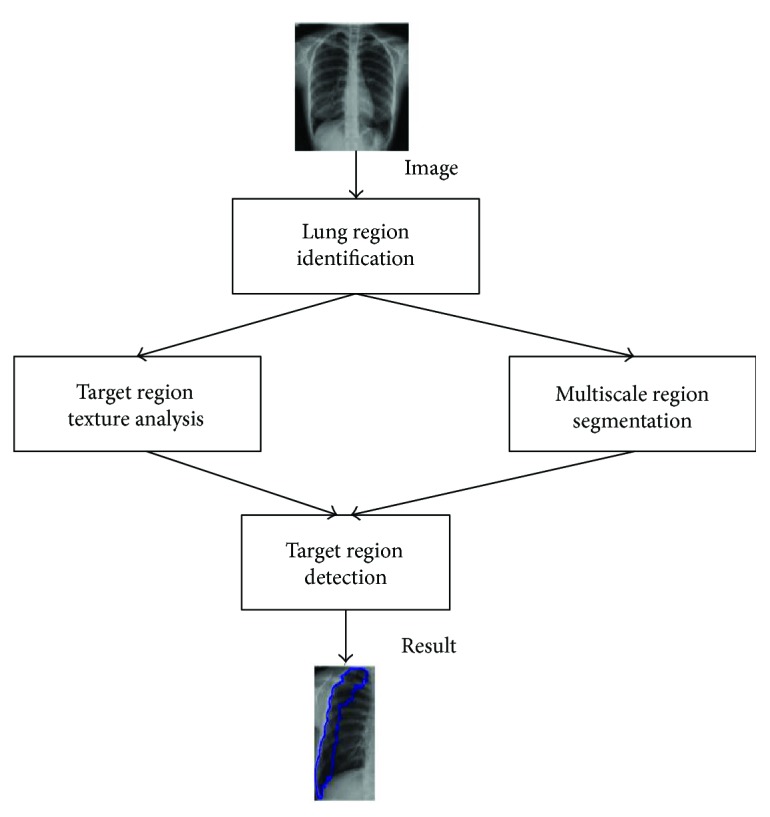
The flowchart of the proposed multiscale intensity texture segmentation.

The multiscale intensity texture segmentation algorithm is composed of lung object identification, target region texture analysis, multiscale region segmentation, and target region detection from chest X-ray images. The lung object identification processes the original image to discover the global lung regions. The target region texture analysis processes the global lung regions to calculate the texture distribution and defines the initial target regions based on the local binary pattern (LBP) [14]. The identification of the multiscale region definition; the smooth, complex regions; and the rib boundaries is performed by the different direction intensity distribution of global lung regions. Finally, the target region detection processes the initial target regions and results of multiscale region segmentation to estimate the relationships and to detect the final target regions. The flowchart of the proposed multiscale intensity texture segmentation is shown in Figure 6.

### 3.2. Identification of Lung Regions

The lung region identification is used to segment the objective global lung regions from original X-ray chest image. After image loading, the mean intensity values of the original image have to be enhanced to the range of intensity from 0 to 1 based on gamma correction to obtain the useful enhanced image through adjustment of the gamma value [11]. Finally, the enhanced image defines the integrated lung regions via the Otsu algorithm [13] to transform the binary image, and then the lung regions are identified based on 8-connected neighborhood method to find two maximum objects that are able to fill empty spaces within the regions.

### 3.3. Texture Analysis of Abnormal Regions

Radiograph construction according to X-ray radiation and interactions with human tissue are composed of 12 bits per pixel whose value corresponds to 0–4095 [15]. The high-density human tissue, such as the bones and lung, can display light intensity by using absorption spectrum. Reversely, the low-density human tissue, such as pneumothorax, displayed dark intensity using attenuation spectrum. The pixel intensity distribution of the original image between 0 and 4095 has sufficient information to describe human tissues, but that may lead to highlight noise. Based on the reason, the 12 bits of the original image are transformed to 8-bit image to reduce the noise for subsequent processing.

**Figure 7:**
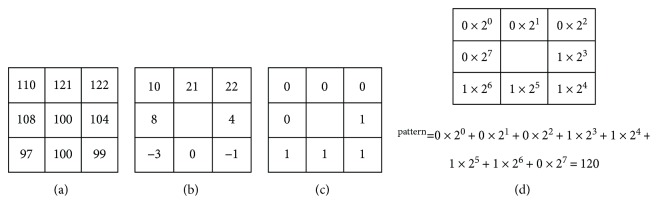
An example of the pattern generation: (a) the original pixel values, (b) difference value of the neighboring point and center point, (c) result of the Win function, and (d) the pattern value of the LBP function.

By following lung region identification, the abnormal regions are recognized by target region texture analysis within the low density of the abnormal region. The proposed target region texture analysis according to the local binary pattern (LBP) defines the smooth and complex regions. LBP image is constructed by *d × d*-sized LBP operator for overlapping and scanning lung regions, then generates pattern histogram from neighboring correlation of patches with *R × R* size for overlapping and scanning lung regions in lung identification. The neighboring correlation of pixel pattern histogram patchPattern_*R*,*d*_{*X_0_*,*X_1_*,…,*X_255_*} after decimal transformation in a moving window is defined as

(5) $$\begin{array}{c} Target\left(patch\left(x_{c},y_{c}\right)\right)=\left\{\begin{array}{ll} 1, & \text{if }{patchPattern}_{R,d}\left(X_{0}\right)=R\times R,\\ 0, & otherwise, \end{array}\right. \end{array}$$

where patch(*x*_*c*_, *y*_*c*_) is a patch with center (*x*_*c*_, *y*_*c*_) and *R* × *R*-sized block, and if the value of patchPattern(*X*_0_) is equal to *R × R*, then Target(patch(*x*_*c*_, *y*_*c*_)) is set to 1; otherwise, Target(patch(*x*_*c*_, *y*_*c*_)) is 0. The center point of 1 in the *R × R* region will be marked as 1. It is judged that the matrix sum of the size *R × R* centering on *x_c_* and *x_y_* is set to 1 if it is equal to *R* × *R* and conversely 0.

(6) $$\begin{array}{c} X_{k}=\overset{R}{\underset{i=1}{\sum }}\overset{R}{\underset{j=1}{\sum }}H\left\{LBP\left(i,j\right)==k\right\},\;k=0,1\ldots ,255, \end{array}$$

where *X_k_* is the number of the *k*th pattern. *i* and *j* are pixel positions of a patch. *H*{*S*} is equal to 1 if *S* is true, and *H*{*S*} is equal to 0 if *S* is false.

(7) $$\begin{array}{c} LBP\left(i,j\right)=\overset{d}{\underset{k=0}{\sum }}2^{k}Win\left(x_{k}-x_{c}\right),\\ Win\left(x_{k}-x_{c}\right)=\left\{\begin{array}{ll} 1, & \text{if }\left|x_{k}-x_{c}\right|\geq 5,\\ 0, & otherwise, \end{array}\right. \end{array}$$

where LBP is a function value where *x_c_* is a center of the *d* × *d*-sized block, and *x_k_* is the neighboring point pixel. Win is a binary thresholding function. An example is shown in Figure 7.

And the formula is similar to (4) but after subtracting the absolute value to see whether greater than or equal to 5 is set to 1 instead of 0.

### 3.4. Multiscale Region Segmentation

After lung region identification, the noise of lung regions is eliminated by Gaussian filter [16] with 5 × 5 mask and standard deviation set as 6 to smooth boundaries of the lung regions. The intensity distributions of horizontal and vertical patterns are constructed from calculating difference values of gradients of horizontal and vertical direction through the Gaussian-filtered image. To segment the regions of different pixel intensity distributions, the constructing patterns of horizontal and vertical direction pattern set are employed for region segmentation.

**Figure 8:**
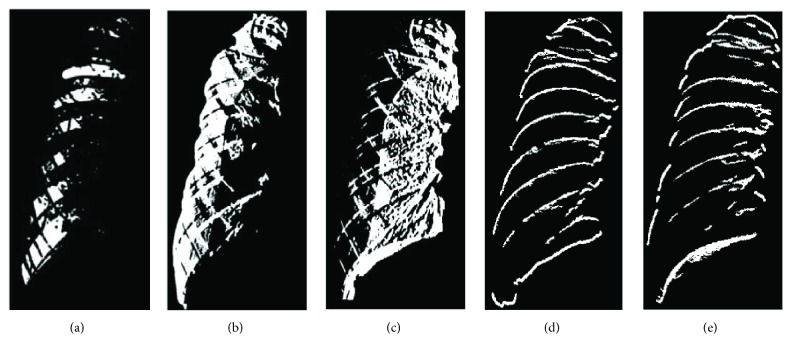
The segmentation results: (a) target region, (b) *H*_1_ intensity distribution pattern set, (c) *H*_2_ intensity distribution pattern set, (d) *V*_1_ intensity distribution pattern set, and (e) *V*_2_ intensity distribution pattern set.

The noise elimination of image is to calculate the difference of intensity between adjacent pixels at horizontal and vertical directions, respectively, for the difference value is able to describe the distribution of pixel intensity. When concerning the horizontal direction, the distribution of pixel intensity is smooth if the difference of value is positive, while the distribution of pixel intensity is complicated if the difference of value is negative. When the distribution of pixel intensity is equal to zero, the distribution of pixel intensity is invariant. At the vertical direction, the distribution of pixel intensity is regarded as the upper boundary of the rib (*V*_1_) if the difference of value is positive while the distribution of pixel intensity is regarded as the lower boundary of the rib (*V*_2_) if the difference of value is negative. The results are shown in Figures 8(d) and 8(c). The different parts of the lung tissue have different pixel intensity distribution.

Next, smooth region, complex region, and rib boundary are determined using the intensity distribution of horizontal scanning and vertical scanning individually, based on the patch of 5 × 5. According to the calculation of difference values, a positive value is set to 1, and a negative value is set to −1. In the horizontal direction, if the number of 1's is larger, the centroid point of the patch is set to 1. Reversely, if the number of −1's is larger, the centroid point of the patch is set to −1. Otherwise, the centroid point of the patch is set to 0. The points set to 1 are defined as *H*_1_ region and the points set to −1 are defined as *H*_2_ region. The results are shown in Figures 8(b) and 8(c). Finally, consider the intersection region of the target and *H*_1_ and the intersection region of the target and *H*_2_. The results which are larger are regarded as smooth regions, and the rest are complex regions. The result of *V*_1_ union *V*_2_ is rib boundary.

### 3.5. Detection of Abnormal Regions

**Figure 9:**
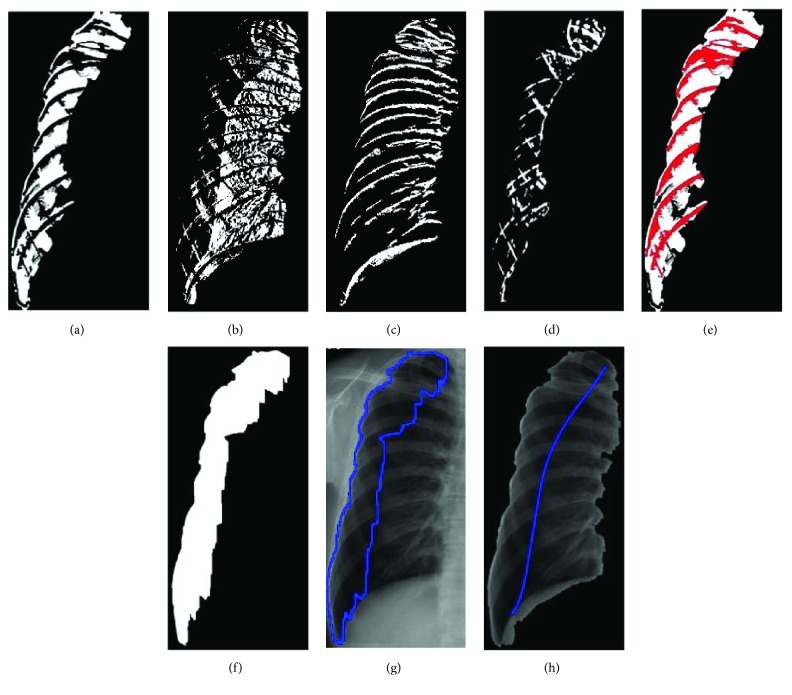
The results of an example of (a) smooth regions, (b) complex regions, (c) rib boundary regions, (d) intersection region between smooth and complex regions, (e)–(f) final segmentation region, and (g)–(h) original image.

After lung regions are determined, the intersection of smooth regions and complex regions will be labeled. Then, more precise smooth regions, constructed by calculating difference value of the standard deviation of the target regions and overlapping region, can be spotted. Firstly, combine the *V*_1_ and *V*_2_ patterns to generate the rib boundaries and remove the rib boundaries for smooth regions and complex regions, as shown in Figures 9(a) and 9(b). Because the rib boundaries do not belong to smooth or complex regions, the process leads to identifying the overlapping regions of smooth and complex regions, as shown in Figure 9(d). For the definition of the new overlapping regions, consider the standard deviation of the overlapping region and the target region. This process calculates the standard deviation for all regions. Because the intensity distribution of the overlapping regions and intensity distribution of the target regions are similar, their standard deviation is naturally similar. Therefore, use the standard deviation of the target region to redefine and determine the overlapping region to be smooth or complex region. If the standard deviation of overlapping region is less than plus or minus 10 percent of the target region, the overlapping region is recognized as the smooth region; otherwise, the overlapping region is removed from the smooth region. Finally, each smooth region is determined as a pneumothorax region. In the rib boundary that is located between the pneumothorax regions, as shown in Figure 9(e), the morphology operations are applied to remove the noises for obtaining final pneumothorax region, as shown in Figure 9(e). The results are then marked on the original image as shown in Figures 9(g) and 9(h).

## 4. Experimental Results

### 4.1. Data Exploratory

32 chest radiographs, including traumatic pneumothorax and spontaneous pneumothorax from 32 patients (age range, 18–65 years) and 10 normal chest radiographs, used in this study are acquired from the Department of Medical Imaging, Chung Shan Medical University Hospital, in Taichung, Taiwan. The dataset was approved by the Institutional Review Board of the Chung Shan Medical University Hospital, in Taichung, Taiwan.

### 4.2. Pneumothorax Prediction Accuracy

The ULBP histogram for 11 × 11 block and 9 × 9 block merged into the final histogram is calculated by using SVM for training and classification. With a sample of 42 images, each image was divided into two lung images (A total of 32 pneumothorax lung cases and 52 normal lung cases). 70% lung cases (36 normal cases and 22 pneumothorax cases) were used for the training phase, while 30% lung cases (16 normal cases and 10 pneumothorax cases) were used for the testing phase. The 5-fold cross-validation result with accuracy (Acc.) variation from 76.9%–88.4% is shown Table 1.

### 4.3. Pneumothorax Segmentation Accuracy

The multiscale intensity texture is calculated by the intensity of each pixel in each block. Finally, the pneumothorax region is detected through standard deviation to evaluate the final recognition of the region of pneumothorax. Three objective evaluations of the accuracy, precision, and sensitivity ratio for comparing the segmentation effects are defined as

(8) $$\begin{array}{c} Accuracy=\frac{TP+TN}{TP+TN+FP+FN},\\ Precision=\frac{TP}{TP+FP},\\ Sensitivity=\frac{TP}{TP+FN}, \end{array}$$

where TP is when both the prediction outcome and the actual value are positive, TN is when both the prediction outcome and the actual value are negative, FP is when the prediction outcome is positive while the actual value is negative, and FN is when the prediction outcome is negative while the actual value is positive.

**Figure 10:**
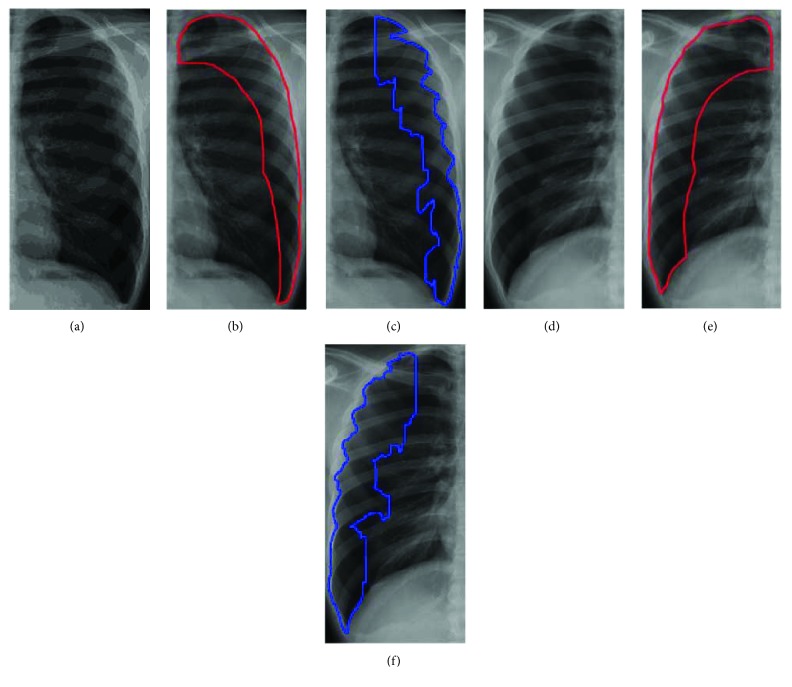
Comparison between the area that is manually depicted of radiologists and the proposed method. (a–c) Segmentation results of the left lung; (d–f) segmentation results of the right lung.

**Figure 11:**
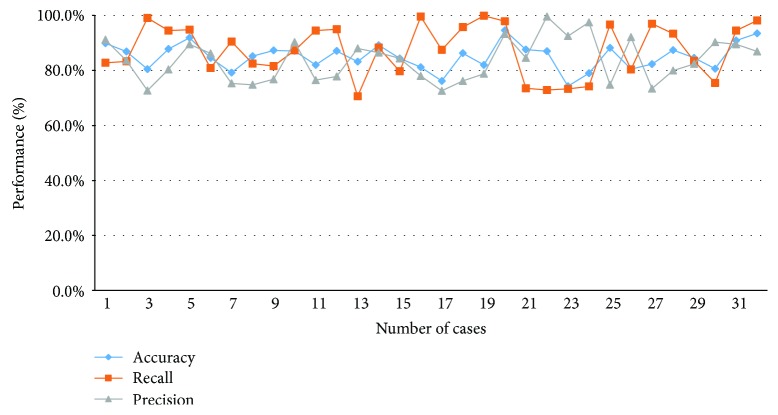
Accuracy, precision, and sensitivity of all dataset image using the proposed method.

Figure 10 shows the comparison between the area that is manually depicted of radiologists and the proposed method. Figures 10(a) and 10(d) are original images, (b) and (e) are pneumothorax regions by manual of radiologists, and (c) and (f) are pneumothorax regions by the proposed method. The accuracy, precision, and sensitivity of all dataset images by using the proposed method are shown in Figure 11.

### 4.4. Comparisons of the Proposed Method

From the experimental results in Figure 11, the accuracy is affected to discern the region between smooth and complex areas in different block sizes. The block sizes of different judgments of the image intensity provide different information; the segmentation will be different in the result. In particular, the image intensity at the boundary and in the vicinity of the clavicle is relatively complicated. In Table 2, the average accuracy of the 5 × 5 block compared to the 11 × 11 block decreases from 85.8% to 81.1%. However, the block size is too small to be used because the region will reveal too little information and then the pneumothorax region cannot be accurately judged. And the block size will increase when the precision increases, but the sensitivity reduces for the evaluation of the precision; it referenced FP and TP, and the sensitivity referenced FN and TP. In the results, when the size of the selected block is larger, the FP decreases but the FN increases. In conclusion, the final cutout pneumothorax region provides a better result for 5 × 5 size of the patch.

## 5. Conclusion

The primary method in the paper is to segment the lung in the abnormal region through multiple overlapping blocks. The abnormal region is found by texture transformed from computing multiple overlapping blocks. Finally, this method effectively analyses lung diseases of the area in the chest X-ray image and improves the possible diagnosis of the missing problem of the pneumothorax area. This increases the efficiency for physicians to assess the extent of the treatment of pneumothorax, so as to support the radiologist to reduce workload.

This study presents a novel framework for automatic pneumothorax detection in CXRs. The texture analysis is based on intensity and gradient for pneumothorax detection. The pneumothorax case was a difficult judgment when pneumothorax region is extremely stenotic and close to the chest boundaries. In addition, pixels located near the chest boundaries tend to have less discriminative texture on image indication, because the bones and pleura existed in obvious edges, which reduced their correspondence of textures. Consequently, the texture characteristic in chest boundaries area is not as prominent as in the inner lung region. Discrimination in different lung regions and adding the texture weight may be the future research focus. The segmentation can increase the accuracy rate for the segmentation of pneumothorax region.

## Figures and Tables

**Table 1:** 5-fold cross-validation result.

Fold	1	2	3	4	5	Acc.
Acc.	76.9%	88.4%	88.4%	80.7%	76.9%	82.2%

**Table 2:** Comparisons of the accuracy, precision, and sensitivity for different patch sizes of the proposed method.

Patch size	Accuracy	Precision	Sensitivity
5 × 5	85.8%	83.6%	87.4%
7 × 7	84.1%	84.4%	85.5%
9 × 9	83.2%	85.1%	82.3%
11 × 11	81.1%	86.2%	81.6%
